# Intragenic *ERG* Deletions Do Not Explain the Biology of *ERG*-Related Acute Lymphoblastic Leukemia

**DOI:** 10.1371/journal.pone.0160385

**Published:** 2016-08-05

**Authors:** Eliska Potuckova, Jan Zuna, Lenka Hovorkova, Julia Starkova, Jan Stary, Jan Trka, Marketa Zaliova

**Affiliations:** 1 Childhood Leukaemia Investigation Prague (CLIP), Department of Paediatric Haematology and Oncology, 2nd Faculty of Medicine, Charles University and University Hospital Motol, Prague, Czech Republic; 2 Department of Paediatric Haematology and Oncology, 2nd Faculty of Medicine, Charles University and University Hospital Motol, Prague, Czech Republic; Pennsylvania State University, UNITED STATES

## Abstract

Intragenic *ERG* deletions occur in 3–5% of B-cell precursor acute lymphoblastic leukemia, specifically in B-other subtype lacking the classifying genetic lesions. They represent the only genetic lesion described so far present in the majority of cases clustering into a subgroup of B-other subtype characterized by a unique gene expression profile, probably sharing a common, however, not yet fully described, biological background. We aimed to elucidate whether *ERG* deletions could drive the specific biology of this *ERG*-related leukemia subgroup through expression of aberrant or decreased expression of wild type *ERG* isoforms. We showed that leukemic cells with endogenous *ERG* deletion express an aberrant transcript translated into two proteins in transfected cell lines and that one of these proteins colocalizes with wild type ERG. However, we did not confirm expression of the proteins in acute lymphoblastic leukemia cases with endogenous *ERG* deletion. *ERG* deletions resulted in significantly lower expression of wild type ERG transcripts compared to B-other cases without *ERG* deletion. However, cases with subclonal *ERG* deletion, clustering to the same *ERG* deletion associated subgroup, presented similar levels of wild type *ERG* as cases without *ERG* deletion. In conclusion, our data suggest that neither the expression of aberrant proteins from internally deleted allele nor the reduced expression of wild type *ERG* seem to provide a plausible explanation of the specific biology of *ERG* -related leukemia subgroup.

## Introduction

Acute lymphoblastic leukemia (ALL), the most common malignancy of childhood, is a heterogeneous disease driven by a wide range of primary and secondary genetic aberrations. Intragenic deletions of *ERG* gene (*ERG*del) occur in 3–5% of B-cell precursor (BCP) ALL and are associated with specific clinical features at disease manifestation and during treatment [1, 2]. *ERG*del occurs almost exclusively in “B-other” ALL defined by the absence of classifying primary genetic lesions (*ETV6/RUNX1*, *BCR/ABL1*, *TCF3/PBX1* and *MLL* (*KMT2A*) gene involving fusions, hyperdiploidy and hypodiploidy). Among B-other ALL, *ERG*del is largely found within a specific subgroup defined by a unique gene expression profile (GEP) [3, 4]. We showed previously that *ERG*del may occur at subclonal level and that it may be lost at disease recurrence [2]. These findings suggest that *ERG*del represents a secondary event at least in a proportion of cases. However, any other aberration occurring in a majority of cases from the above mentioned GEP-defined *ERG*-related ALL subtype (potentially defining its common genetic background) has not been identified besides *ERG*del so far. Thus, despite possibly representing a secondary event *ERG*del may still significantly impact the biological character of this subgroup.

*ERG* encodes a transcription factor, a member of the ETS family that regulates many important biological processes. *ERG* is essential for maintenance of hematopoietic stem cells and is supposed to play a role in a lineage development [5, 6]. As a fusion partner of *FUS* gene *ERG* is causally involved in the development of acute myeloid leukemia with the t(16;21) translocation [7]. *ERG* is also strongly implicated in the development of the acute megakaryocytic leukemia in children with constitutional trisomy of chromosome 21 where *ERG* gene resides [8, 9]. Its enforced expression in bone marrow cells induces T-cell and (erythro)megakaryocytic leukemia and blocks B-lineage differentiation at pro-B to pre-B transition in mice [10, 11].

The *ERG* gene encodes several isoforms; two isoforms—ERG2 and ERG3 –are predominantly expressed in hematopoietic tissue [12]. At the protein level these isoforms contain two well-conserved functional domains, PNT and ETS, which participate in protein-protein interactions and the latter mediates DNA binding and transactivation of *ERG* targets [13].

Intragenic deletions of *ERG* affect several exons and result in the loss of the internal part of the protein coding region. Proximally and distally to the site of breakpoints junction unaffected parts of the coding regions are maintained. It was suggested, that using the canonical and alternative translation start sites two different aberrant isoforms might be expressed from the affected allele: 1) prematurely truncated N-terminal protein lacking both functional domains and 2) truncated C-terminal protein with intact ETS domain that could potentially act as a competitive inhibitor of wild type isoforms [4]. Any detailed study supporting this hypothesis has not been published so far. Thus, it still remains elusive whether *ERG*del represents a dominant-negative or potentially gain-of-function aberration. Moreover, it has not been shown whether the loss of one germline ERG allele impacts the expression level of physiological ERG isoforms in *ERG*del-positive ALL.

The aim of our study was to characterize the biological impact of intragenic *ERG* deletions. We focused on the above mentioned aberrant *ERG* isoforms as potential mediators of dominant-negative or gain-of function role of *ERG*del. We have studied their expression at mRNA and protein levels and their sub-cellular localization in model systems in vitro and in primary *ERG*del-positive ALL samples. To address the question of wild type *ERG* levels we analyzed expression of *ERG* in *ERG*del-positive versus *ERG*del-negative ALL patients in the available data from whole genome gene expression profiling study and using quantitave PCR in our independent cohort of patients.

## Methods

### Patients

Biological material from the diagnostic bone marrow aspiration of 46 children with ALL with more than 70% of blasts was used within this study including 9 *ERG*del-positive B-other ALL, 13 B-other ALL with *ERG*del in subclone, 21 *ERG*del-negative B-other ALL, 2 BCP-ALL with hyperdiploidy and 1 *ETV6/RUNX1*-positive ALL. As a part of routine diagnostics immunophenotype, DNAindex, karyotype and presence of *ETV6/RUNX1*, *BCR/ABL1*, *TCF3/PBX1* and *MLL* gene involving fusions were assessed in all samples. ALL cases with B-cell precursor immunophenotype and negative for all above mentioned fusions, hyperdiploidy and hypodiploidy were assigned into B-other ALL subgroup. Copy number aberrations were analyzed in all samples using high density SNP array (HumanOmni Express BeadChip, Illumina, San Diego, USA). Besides SNParray, the presence/absence of *ERG*del was analyzed by PCR as described previously [2]. Patients without *ERG* deletion on SNParray and negative by PCR screening were called *ERG*del negative. Patients with *ERG*del on SNParray and positive by PCR screening were called *ERG*del positive. Patients without *ERG*del on SNParray and positive by PCR screening were called *ERG*del positive in subclone. The study was approved by Institutional Review Board in the University Hospital Motol and a written informed consent was obtained from parents or guardians of all children whose biological material was used in the study in accordance with the Declaration of Helsinki.

### Cell lines

HeLa (human cervical adenocarcinoma), NALM6 and REH (both human B-cell precursor acute lymphoblastic leukemia) cell lines were purchased from German Collection of Microorganims and Cell Cultures (DSMZ-Deutsche Sammlung von Mikroorganismen und Zellkulturen GmbH, Germany). HEK293T (human embryonic kidney) cell line was kindly provided by Dr. Alberich-Jorda (IMG ASCR; Prague, Czech Republic).

### Cloning of *ERG* isoforms

**Fig 1:**
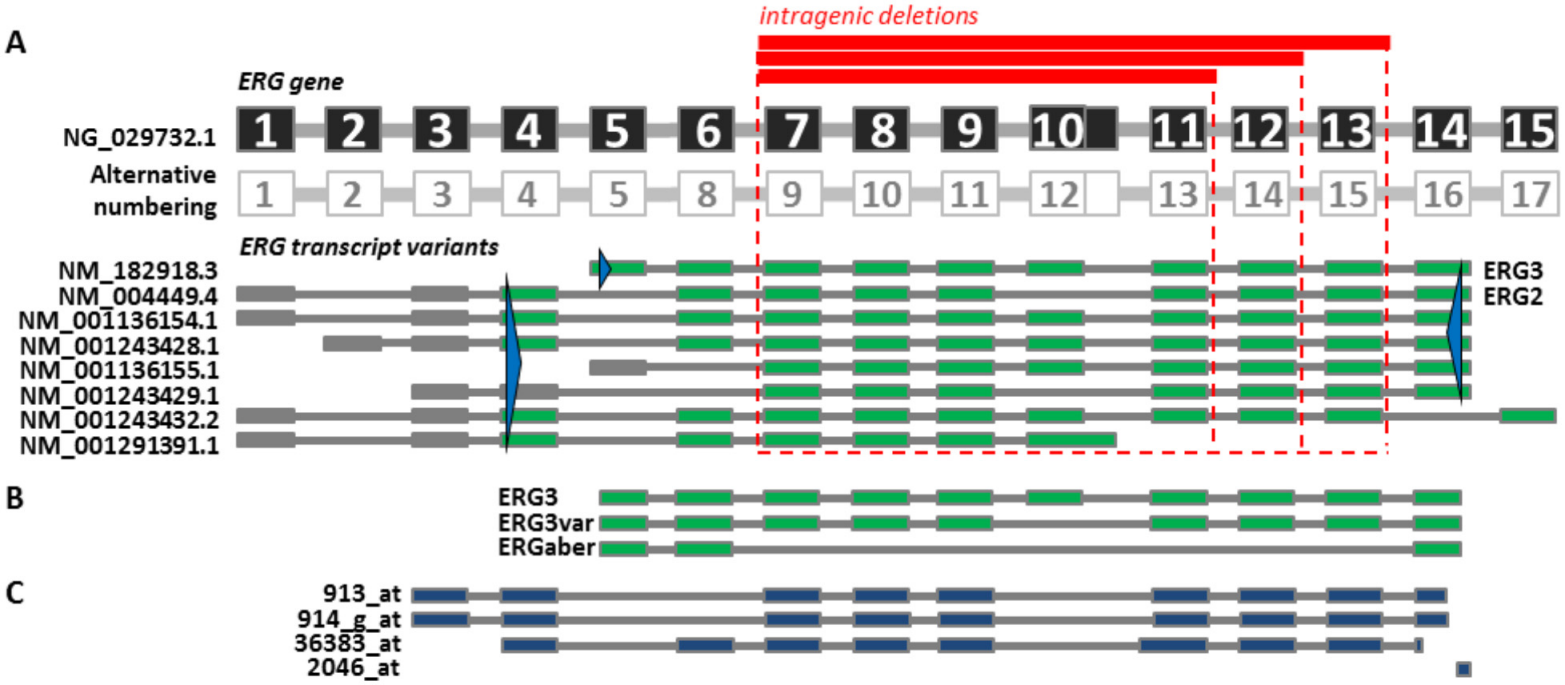
ERG gene and its transcript variants. (A) Schematic representation of *ERG* gene and its transcript variants according to the current reference sequences available in NCBI Reference Sequence Database. Exons (represented by boxes) are numbered from 5’ to 3’ within the ERG gene (RefSeq ID NG_029732.1). Alternative exon numbering used by Owczarek et al. and by Bohne et al.[12, 14] is also shown. Transcript variants are annotated with RefSeq accession numbers and names for the encoded protein isoforms commonly used in the literature (ERG3 for NM_182918.3 and ERG2 for NM_004449.4); coding exons are in green, non-coding in grey. Recurrent types of intragenic deletions are displayed in red. Forward and reverse primers (blue triangles) used for amplification of full-length coding regions are positioned across all variants which they can theoretically amplify. (B) Schematic representation of three successfully amplified and cloned *ERG* transcript variants. (C) Probe sets from HG_U95Av2 Affymetrix gene expression array annotated to *ERG* gene exons. The scheme of probe sets mapping was adapted from UCSC Genome Browser (Kent WJ, Sugnet CW, Furey TS, Roskin KM, Pringle TH, Zahler AM, Haussler D. The human genome browser at UCSC. Genome Res. 2002 Jun;12(6):996–1006).

Total RNA from NALM6 and REH cell lines and from primary diagnostic ALL samples was transcribed into cDNA employing Cloned AMV First-Strand cDNA Synthesis Kit (Thermo Fisher Scientific) with Oligo (dT)20 as a primer. The whole coding regions of wild type ERG2 and ERG3 and of aberrant *ERG* isoform(s), potentially transcribed from internally deleted allele, were amplified from cDNA by PCR using primers annealing to regions involving start codons and stop codons of these ERG isoforms (see Fig 1A for schematic primer positions). Amplified coding sequences were cloned into pcDNA3.1 (kindly provided by Dr. Anthony Ford, Institute of Cancer Research, UK) vector. The primer sequences are listed in Table A in S1 File.

### In vitro transcription and translation assay (T/T assay)

N-terminal and C-terminal ERGaber proteins (ERGaber-C, ERGaber-N) were synthesized in vitro using TNT^®^ Quick Coupled Transcription/Translation Systems (Promega, Wisconsin, USA) according to manufacturer’s protocol. Two separate segments of ERGaber coding sequence encoding ERGaberN and ERGaberC amplified using primers listed in Table B in S1 File served as templates.

### Transfection of HeLa and HEK293T cells

HeLa and HEK293T cells were transfected using Lipofectamine2000 (Thermo Fisher Scientific) reagent according to manufacturer’s instructions. Forty-eight hours after transfection cells were harvested and used for protein analysis.

### Western blot

Nuclear and cytoplasmic protein lysates from HeLa and HEK293T cells transfected with ERGaber-pcDNA3.1, ERG3var-pcDNA3.1 or ERG3-pcDNA3.1, from NALM6 cells and from primary ALL samples were prepared using NE-PER Nuclear and Cytoplasmic Extraction Reagents (Thermo Fisher Scientific) supplemented with Complete Protease Inhibitor Coctail (Roche, Switzerland) according to manufacturer’s instruction. Primary and secondary antibodies used for proteins detection are listed in Table C in S1 File.

### Confocal microscopy

HeLa and HEK293T adhered to sterile cover slips were transfected with pcDNA3.1 based *ERG* constructs as described above. Forty-eight hours after transfection cells were fixed and stained by primary and secondary antibodies (listed in Table C in S1 File) and by DAPI (Thermo Fisher Scientific). Microscope slides were inspected using Leica DMi8 inverted microscope equipped with TCS SP8 confocal system and Leica Application Suite X software (Leica Microsystems, Germany).

### Analysis of ERG expression from publically available genome-wide gene expression profiling data

Gene expression data of 327 ALL cases analyzed on HG_U95Av2 oligonucleotide array by Yeoh et al. were downloaded from http://www.stjuderesearch.org/ALL1 and normalized as described by Yeoh et al. [15]. Data for four probe sets mapping to *ERG* exons were extracted from normalized dataset.

### Quantification of physiological *ERG* isoforms by PCR

The expression level of physiological *ERG* isoforms with and without *ERG* exon 10 and *ABL1* transcript (used as an endogenous control to normalize cDNA concentration) was measured by quantitative reverse transcription PCR (qRT-PCR). The *ABL1* transcript was measured as described previously [16]. Sequences of primers and hydrolysation probe used to quantify *ERG* isoforms are listed in Table D in S1 File. Serial dilutions of plasmid calibrators containing the sequences of measured transcripts (ipsogen ABL Control Gene 3 Standards, Qiagen, USA; in-house established plasmid calibrators for *ERG* detection systems) were used for standard curve construction and absolute quantification.

Detailed methods are available in S1 File.

## Results

### Aberrant ERG transcript is expressed in ERGdel-positive ALL cases

**Fig 2:**
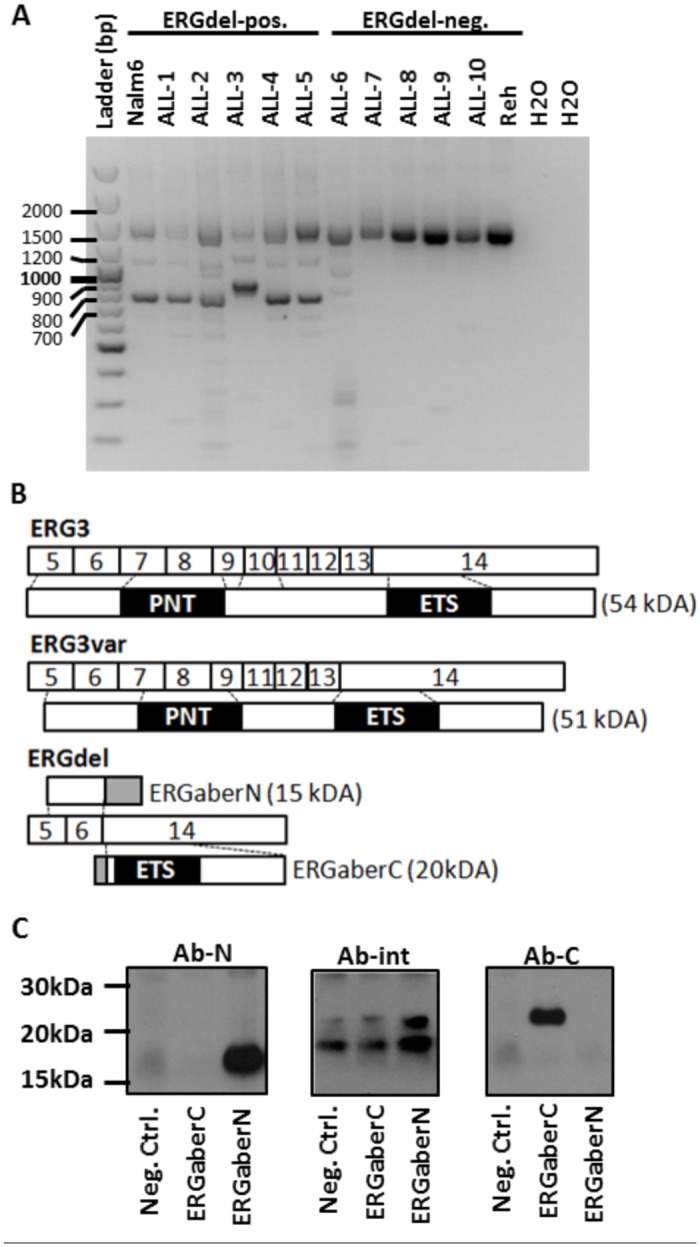
Amplification, cloning and translation of ERG isoforms. A) Analysis of PCR-amplified *ERG* isoforms by electrophoresis on agarose gel. PCR products with length corresponding to ERG3 coding region (1458bp) were present in all samples. Calculated length of amplified coding region of predicted aberrant *ERG* isoforms was 775bp in NALM6 and ALL samples 1,2,4 and 5 (bearing the deletion of *ERG* exons 7–13) and 880bp in ALL sample 3 (bearing the deletion of *ERG* exons 7–11). PCR products corresponding to predicted aberrant *ERG* isoforms were present in all *ERG*del-positive samples and in none *ERG*del-negative samples (B-other cases: ALL-6 and ALL-7, hyperdiploid cases: ALL-8 and ALL-9, *ETV6/RUNX1*-positive case—ALL-10). (B) Schematic representation of *ERG* transcript variants cloned from NALM6 and of predicted encoded proteins. Regions encoded by alternative frames are displayed in grey. (C) Analysis of ERGaber proteins synthesized from PCR-prepared DNA templates by in vitro transcription/translation (T/T) assay. Proteins were detected by western blot using following antibodies: Anti-ERG antibody EPR3863 (Ab-N), Erg-1/2/3 Antibody C-17 (Ab-int), Erg-1/2/3 Antibody C-20 (Ab-C). In vitro T/T reaction without any DNA template served as a negative control (Neg. Ctrl.). ERGaberN was detected by Ab-N only and ERGaberC by Ab-C only. Ab-int showed only unspecific binding to proteins present in reticulocyte lysate used for T/T assay.

Since *ERG*del does not affect *ERG* promoter and transcription start site, an aberrant transcript from the affected allele can be theoretically expressed. To verify this hypothesis we performed PCR using cDNA from NALM6 cell line (bearing *ERG*del) and primary ALL samples with primers enabling simultaneous amplification of complete coding sequence of ERG2 and ERG3 (expressed from the wild type allele) and of potential aberrant transcripts (expressed from the allele affected by deletion) (Fig 1A). While we did not get reproducible PCR products using primers annealing to exon 4 (data not shown) we got clear PCR products corresponding to ERG3 using primer annealing to *ERG* exon 5 in all analyzed samples (Fig 2A). Additional shorter PCR products (with length corresponding to the predicted aberrant transcripts) were obtained in all *ERG*del-positive ALL cases (n = 5) and in NALM6 cell line but not in any *ERG*del-negative ALL case (n = 5) or in REH cell line (not bearing *ERG*del). Subsequent cloning and sequencing of PCR products confirmed the presence of 3 different isoforms—ERG3, ERG3var and ERGaber (depicted on Figs 1B and 2B)—which were further studied in subsequent experiments. In comparison to ERG3, ERG3var lacks exon 10 encoding part of the protein between the PNT and ETS domains. The short length of this exon (72bp) prevented detection of ERG3var as a unique band on routine agarose gel, probably due to co-migration with ERG3 (1458bp). ERG3var does not correspond to any of the physiological transcripts described in NCBI Reference Sequence Database, however, it was already described by Bohne et al. (who assigned it ERG3Δex12 according to the alternative exon numbering displayed in Fig 1A) in leukemic samples as well as in healthy hematopoietic tissue suggesting its physiological nature [12]. ERGaber transcripts lack exons affected by the genomic deletion and thus probably represent aberrant isoform expressed from the affected allele.

### ERGaber transcript is translated into ERGaberN and ERGaberC proteins in vitro

ERGaber transcript involves only three exons: 5, 6 and 14. Due to a disruption of canonical reading frame at exon 6/14 junction (Fig A in S1 File) the translation from canonical start codon (located in exon 5) theoretically results in a prematurely truncated protein—ERGaberN (Fig 2B; Fig A panels B and C in S1 File). This protein (of predicted molecular weight 15kDa) lacks PNT and ETS domains, its N-terminal part is homologous to N-terminus of ERG3 and it contains 57 C-terminal amino acids encoded by alternative reading frame of exon 14. In silico analysis showed a single start codon within an alternative reading frame of exon 6 which could theoretically initiate translation of another truncated protein—ERGaberC—of predicted molecular weight 20kDa (Fig 2B; Fig A panels D and E in S1 File). ERGaberC has an altered N-terminus, while its C-terminus is homologous to the C-terminus of ERG3 and contains complete ETS domain. Using PCR-prepared templates corresponding to complete coding sequences of ERGaberN and ERGaberC we successfully translated both proteins in vitro (Fig 2C). We tested 3 different antibodies for detection of these proteins by western blot: Ab-N, Ab-int and Ab-C recognizing epitopes formed by N-terminal, internal and C-terminal amino acids, respectively. While we detected ERGaberN using Ab-N and ERGaberC using Ab-C, the Ab-int antibody did not detect any of these proteins suggesting they both lack its epitope.

### ERGaberC is expressed at low level and has different sub-cellular localization compared to ERGaberN in cell line model

**Fig 3:**
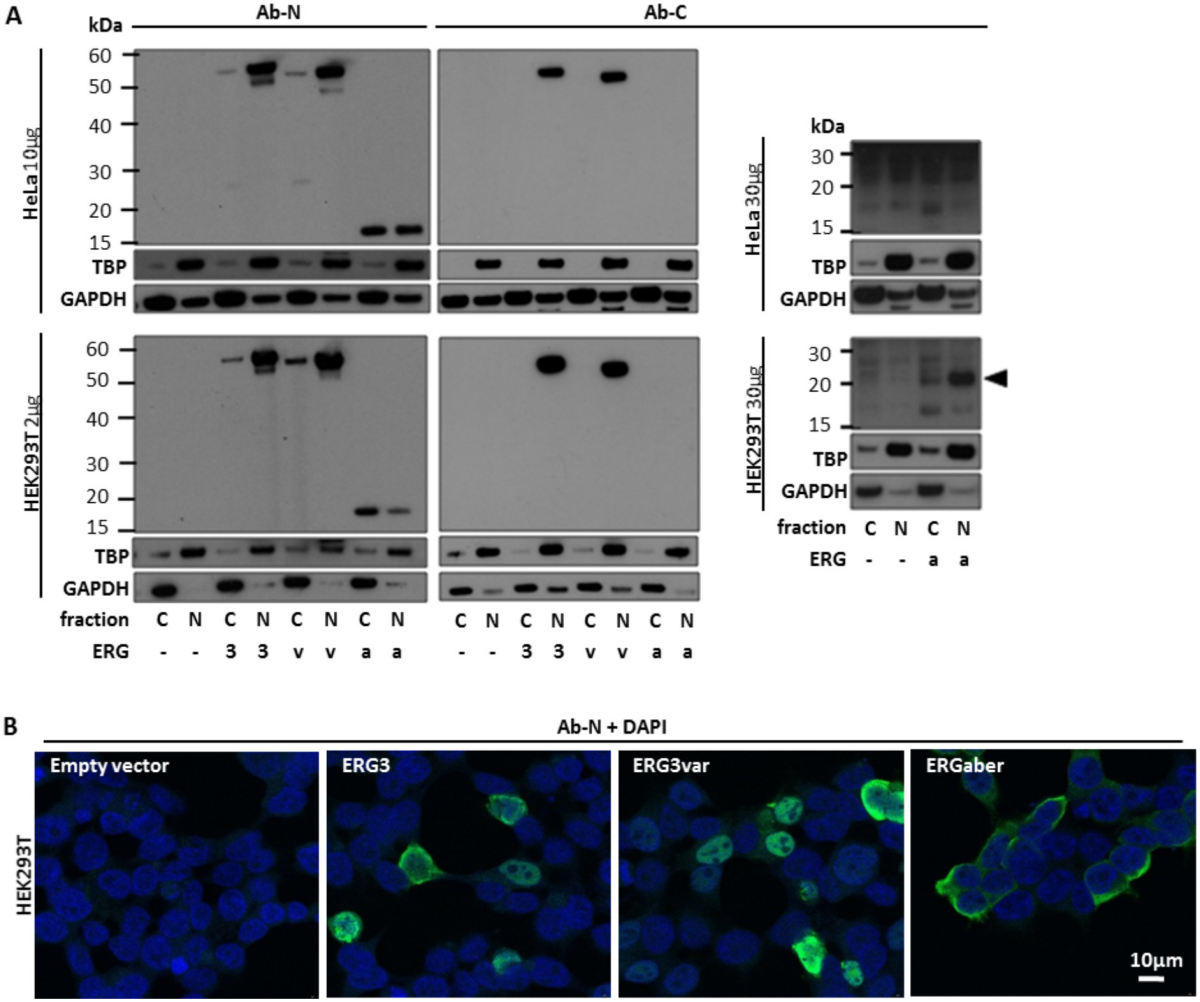
Analysis of subcellular localization of *ERG* isoforms by western blot and confocal microscopy. (A) Protein lysates from HeLa and HEK293T cells (10μg and 2μg, respectively) transiently transfected by ERG3 (3), ERG3var (v) and ERGaber (a) in pcDNA3.1 vector or by empty vector (-) were analyzed by western blot to determine subcellular localization of individual ERG isoforms (left panel). ERG3 and ERG3var were detected dominantly in the nuclear fraction of protein lysate (N) in both cell lines, while ERGaberN was found in both cytoplasmic (C) and nuclear fractions in HeLa cells and dominantly in cytoplasmic fraction in HEK293T cells. ERGaberC was not detected in any cell line using these protein loads. Using higher load of protein lysate (30μg), sensitive visualization kit and longer exposition to X-ray films ERGaberC was detected in nuclear fraction of HEK293T but not HeLa cells (right panel, black arrow points to corresponding protein band). TBP and GAPDH proteins were used to control protein load and separation of cellular fractions. (B) HEK293T cells were transiently transfected by ERG3, ERG3var and ERGaber isoforms in pcDNA3.1 vector or by empty vector. Forty-eight hours after transfection the presence and the subcellular localization of *ERG* isoforms was analyzed by confocal microscopy using Ab-N antibody. Nuclei were stained by DAPI. The scale bar represents 10μm.

We transiently transfected plasmid vectors with coding sequences of ERG3, ERG3var and ERGaber into HeLa and HEK293T cells to study their sub-cellular trafficking. Using western blot on sub-cellular fractions and confocal fluorescence microscopy we found ERG3 and ERG3var to localize dominantly in nucleus (Fig 3 and Fig B in S1 File). ERGaberN was detected both in nucleus and in cytoplasm in HeLa cells, while it was mainly present in cytoplasm in HEK293T cells (Fig 3 and Fig B in S1 File). We were not able to visualize ERGaberC in any of these cell lines using confocal fluorescence microscopy (data not shown). Using western blot we detected ERGaberC in nuclear fraction of HEK293T cells (but not in cytoplasm) only when increasing the protein load 15 times in comparison with the load sufficient for successful detection of ERG3, ERG3var and ERGaberN (Fig 3).

### ERGaber proteins were not detected in NALM6 cell line or in primary *ERG*del-positive ALL

**Fig 4:**
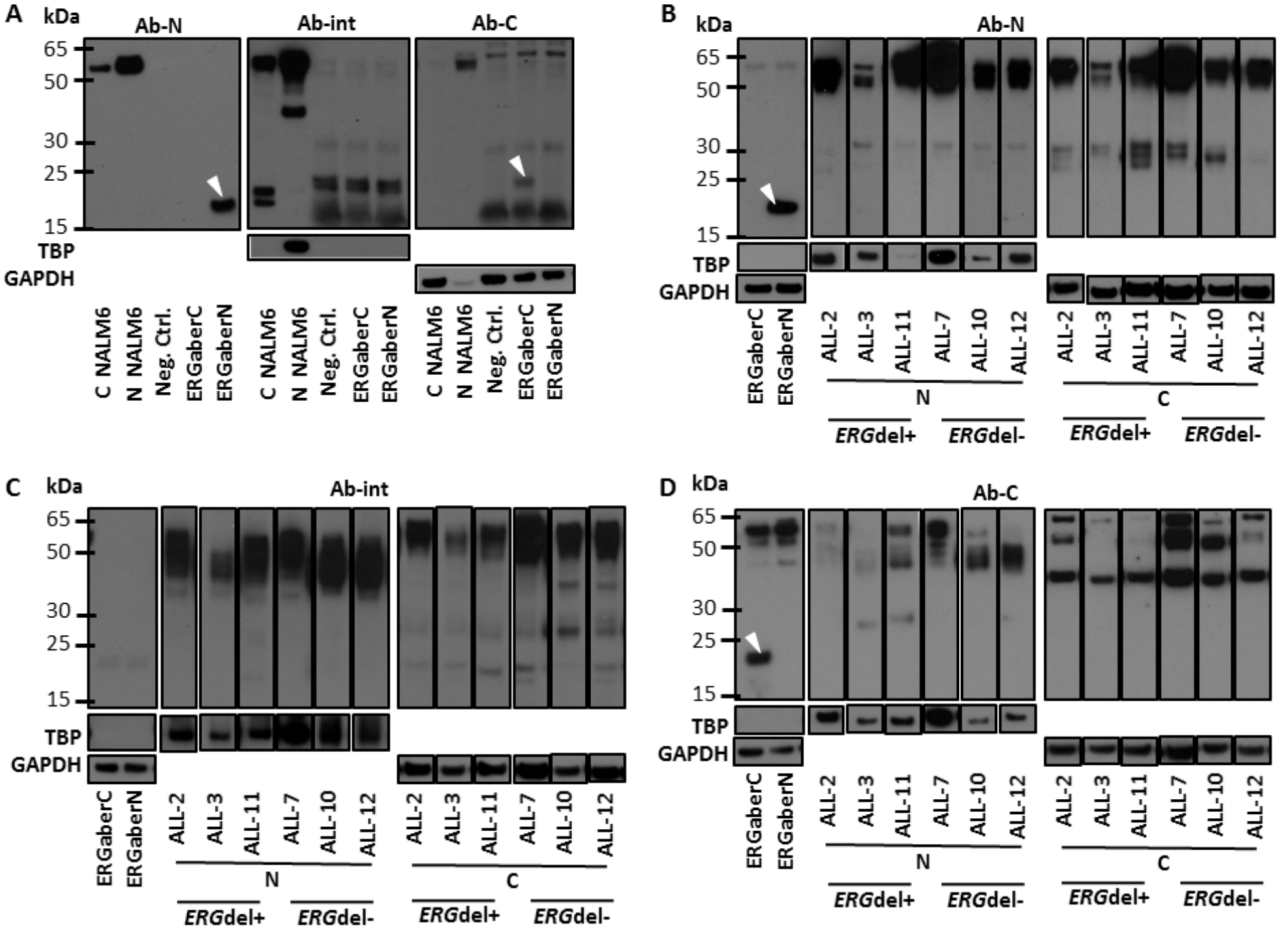
ERG protein expression in NALM6 cells and in patient samples. The presence of ERGaberN and ERGaberC proteins in nuclear (N) and cytoplasmic (C) fractions of protein lysates from NALM6 cell line (panel A), ERG-del-positive (n = 3) and ERGdel-negative (two B-other– ALL-7, ALL-12; one ETV6/RUNX1-positive—ALL-10) ALL cases (panel B, C and D) was analyzed by western blot. ERGaberN and ERGaberC proteins synthesized by T/T assay served as positive controls (corresponding bands are highlighted by white arrows). Any protein bands possibly corresponding to ERGaberN or ERGaberC were detected neither in nuclear nor in cytoplasmic protein fractions of NALM6 nor in ALL samples. Two bands were detected in cytoplasmic fraction of NALM6 cells (within 15-25kDA molecular weight region) using Ab-int antibody which, however, lacks reactivity against ERGaberN and ERGaberC. TBP and GAPDH proteins were used to control protein load and separation of cellular fractions. Protein loads: 10μg of nuclear and cytoplasmic proteins of NALM6; 10–20μg of nuclear and 25–43μg of cytoplasmic proteins of ALL cases. Panels B, C and D: individual lanes were cut from the scans of X-ray films and grouped. Full scans of X-ray films are available in Fig C in S1 File. These contain additional lanes which were excluded from the analysis because of protein degradation or subclonality of *ERG*del.

To confirm our findings from artificial cell line based models we analyzed leukemic cells harboring endogenous *ERG*del. Using Ab-N and Ab-C antibodies we were unable to detect any of the ERGaber proteins either in NALM6 cells or in primary *ERG*del-positive ALL samples (Fig 4). Clappier et al. previously demonstrated the presence of short ERG isoform in primary *ERG*del-positive ALL using Ab-int. Although we showed that this antibody is not capable to detect ERGaber proteins characterized in our in vitro assays, we employed it in our western blots as well. While Ab-int detected physiological *ERG* isoforms, we did not find any short ERG isoform present specifically in *ERG*del-positive compared to *ERG*del-negative primary ALL samples. There were 2 bands in cytoplasmic protein fraction of NALM6 cell line (within the region of 15-25kDa) detected only by Ab-int but not by the other antibodies. These bands may represent some short *ERG* isoforms, however, since we showed that Ab-int detects physiological *ERG* isoforms but neither ERGaberN nor ERGaberC, these short isoforms probably contain amino acids encoded by region that is not involved in ERGaber transcript and, thus, they are rather expressed from the wild type *ERG* allele.

### Expression of physiological *ERG* transcripts is lower in *ERG*del-positive compared to *ERG*del-negative B-other ALL

To evaluate expression of wild type *ERG* that could be potentially influenced by lower gene-dosage in ALL cases bearing monoallelic *ERG* deletion we analyzed expression of physiological *ERG* isoforms at mRNA level.

**Fig 5:**
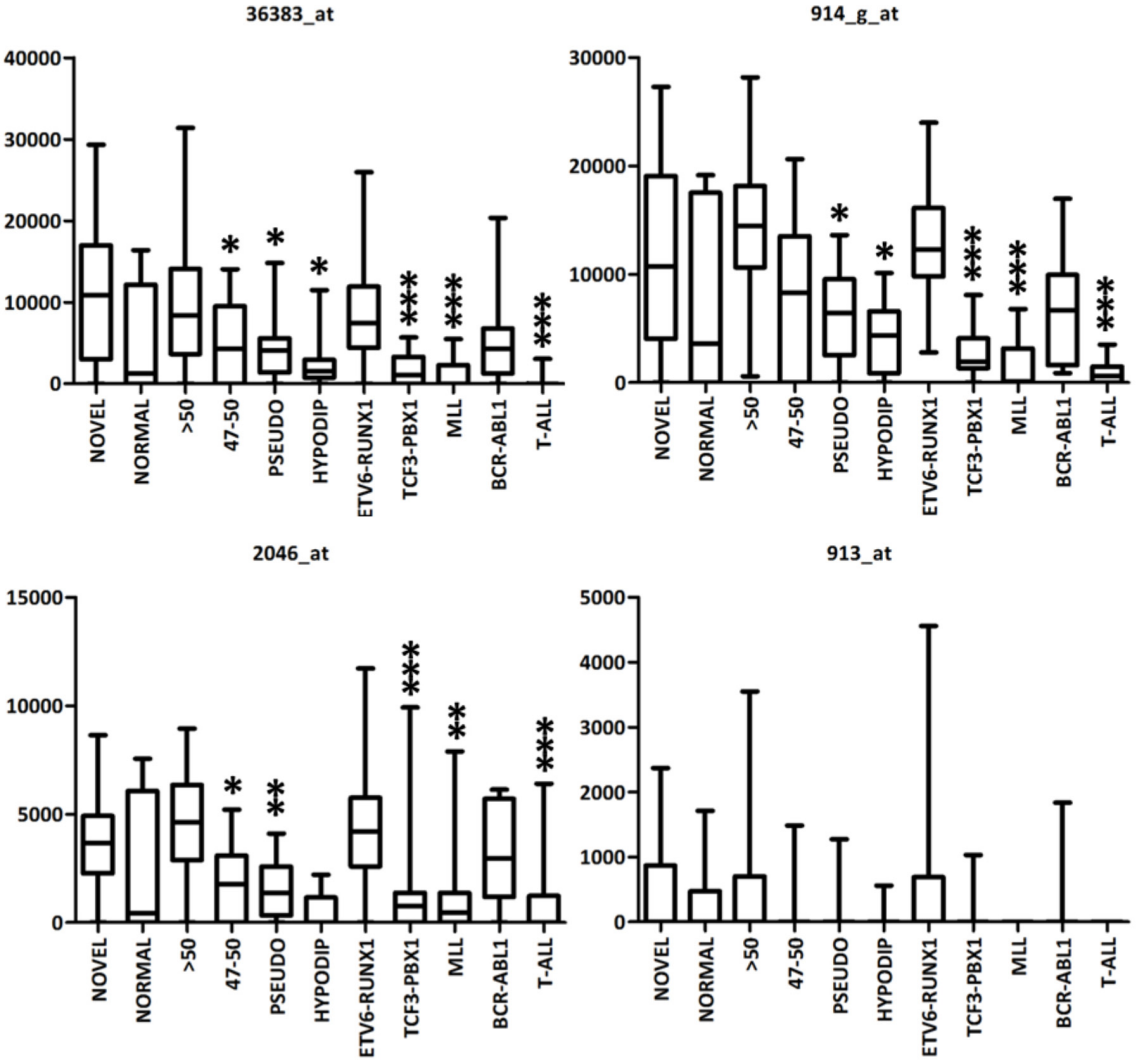
Expression of probe sets annotated to *ERG* coding region in ALL subgroups. Normalized expression levels (Y axis) of 4 probe sets annotated to *ERG* exons in 11 ALL subgroups (X axis): *ERG*del enriched B-other ALL (NOVEL, n = 14), B-other ALL with normal karyotype (NORMAL, n = 11), high hyperdiploid ALL (>50, n = 63), ALL with 47–50 chromosomes (47–50, n = 21), ALL with pseudodiploid karyotype (PSEUDO, n = 25), ALL with <47 chromosomes (HYPODIP, n = 9), *ETV6-RUNX1*-positive ALL (*ETV6-RUNX1*, n = 79), *TCF3-PBX1*-positive ALL (*TCF3-PBX1*, n = 27), *MLL*-rearranged ALL (*MLL*, n = 20), *BCR-ABL1*-positive ALL (*BCR-ABL1*, n = 15) an T-ALL (n = 43). Boxes extend from the 25th to 75th percentiles, the lines in the middle represent medians, and the whiskers extend to minimal and maximal values. ALL subgroups labeled with asterisks have significantly different expression compared to *ERG*del associated B-other ALL subgroup: * −0.01 < p ≤ 0.05; ** −0.001 < p ≤ 0.01; *** −p ≤ 0.0001 (Mann-Whitney U test).

We first used publically available data from whole genome gene expression profiling to compare *ERG* expression across several ALL subgroups including “novel” ALL subgroup highly enriched for *ERG*del-positive cases (*ERG-*related ALL subgroup). In total, four probe sets from the data by Yeoh et al. map to the *ERG* exons (Fig 1C). The 913_at probe set was expressed at very low levels in general; the expression pattern of remaining three probe sets was similar and the *ERG-*related ALL subgroup had either significantly higher or comparable expression in comparison to other ALL subgroups (Fig 5). Unfortunately, all four probe sets include probes mapping to the exon(s) involved in ERGaber transcript (expression of which we confirmed in primary *ERG*del-positive ALL as described above). Thus, the expression of these probe sets in *ERG*del-positive cases probably reflects sum of expression from both physiological and aberrant isoforms.

**Fig 6:**
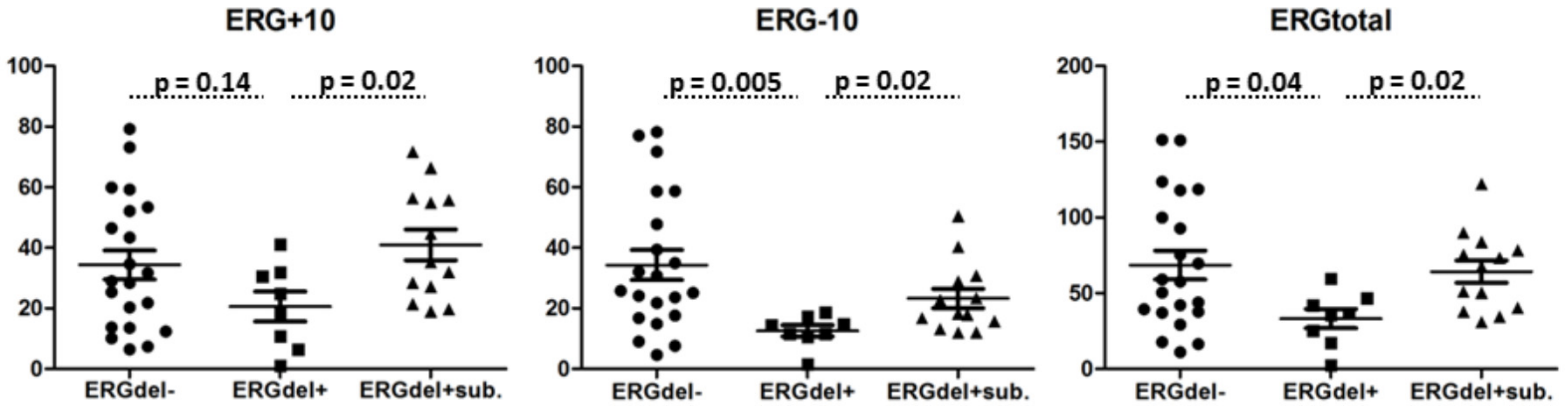
Expression of physiological *ERG* isoforms in ALL subgroups. Normalized expression levels (Y axis) of physiological *ERG* isoforms with (ERG+10) and without (ERG-10) exon 10 and their sum (ERGtotal) in 21 *ERG*del-negative B-other ALL cases (*ERG*del-), 8 *ERG*del-positive B-other ALL cases (*ERG*del+) and 13 B-other ALL cases with *ERG*del at sublocnal level (*ERG*del+sub). Horizontal lines represent means (ERG+10: 34.4±21.4, 20.6±13.0 and 41.0±17.6 for *ERGd*el-, *ERG*del+ and *ERG*del+sub, respectively; ERG-10: 34.3±22.1, 12.6±4.9 and 23.3±11.2 for *ERG*del-, *ERG*del+ and *ERG*del+sub, respectively; ERGtotal: 68.7±42.5, 33.2±16.7 and 64.3±25.5 for *ERG*del-, *ERG*del+ and *ERG*del+sub, respectively). Mann-Whitney U test was used to compare two groups.

We have designed two qRT-PCR systems specific for physiological *ERG* isoforms with and without *ERG* exon 10 and used them to measure their expression in an independent cohort composed of 42 B-other ALL cases. Besides *ERG*del-negative (n = 21) and *ERG*del-positive (n = 8) cases we also included cases with intragenic *ERG* deletion present at subclonal level (*ERG*del+sub, n = 13). These cases belong to the same GEP-defined ALL subgroup as *ERG*del-positive cases, however, they still have two intact *ERG* alleles in the vast majority of leukemic cell population. Although the expression levels varied considerably within the analyzed subgroups, we found significantly lower total *ERG* expression in *ERG*del-positive compared to *ERG*del-negative and *ERG*del+sub cases (*ERG*del-positive vs *ERG*del-negative p = 0.04; *ERG*del-positive vs *ERG*del+sub. p = 0.02; Fig 6). The expression level of *ERG* isoforms without *ERG* exon 10 was significantly lower in *ERG*del-positive compared to both *ERG*del-negative and *ERG*del+sub cases (p = 0.005 and p = 0.02, respectively). The expression level of *ERG* isoforms with *ERG* exon 10 was significantly lower in *ERG*del-positive compared to *ERG*del+sub cases (p = 0.02) and tended to be lower also when compared to *ERG*del-negative cases, however, this trend did not reach a statistically significant level (p = 0.14). The expression levels in *ERG*del+sub did not differ significantly when compared to *ERG*del-negative cases. The ratio of exon 10 involving to exon 10 lacking isoforms did not differ significantly between ERGdel-positive compared to ERGdel-negative and ERGdel+sub cases (p = 0.3 for both comparisons). Any functional difference between exon 10 involving versus exon 10 lacking isoforms has not been described in BCP-ALL so far and we presume that their function is highly analogous. Thus, we consider the level of the total ERG expression, which was approximately twofold in *ERG*del-negative and *ERG*del+sub cases compared to *ERG*del-positive cases (68.7±42.5 and 64.3±25.5 vs 33.2±16.7), to represent the most relevant measure of wild type ERG expression.

## Discussion

To better understand the potential biological role of intragenic *ERG* deletions (which associate with specific clinical features and characterize a biologically unique *ERG*-related ALL subgroup) we aimed to study two possible primary consequences of this aberrations: 1) the expression of aberrant dominant-negative (or gain-of-function) gene products and/or 2) gene dosage dependent reduction of physiological gene products.

We showed that *ERG*del-positive ALL (both primary ALL cases and cell line) express an aberrant ERG transcript which lacks all genomically deleted exons and which is not present in *ERG*del-negative ALL and, thus, is probably transcribed from the affected allele. We have cloned its complete coding sequence and showed that it is translated into two aberrant proteins in transfected cell lines– ERGaberN and ERGaberC. While ERGaberN (lacking both conserved domains) was dominantly trafficked into cytoplasm, ERGaberC (possessing an intact ETS domain) was retained in nucleus; however, it was expressed at significantly lower level compared to ERGaberN in transfected cells. Moreover, we subsequently detected neither ERGaberN nor ERGaberC in primary leukemic cells with endogenous *ERG* deletion despite the reactivity of the used antibodies against both aberrant proteins was reliably proven in our in vitro experiments. This data suggest that despite there is the aberrant transcript expressed, the predicted aberrant proteins are produced at very low levels compared to physiological *ERG* isoforms or are not expressed at all.

We found a significantly lower expression of physiological ERG transcripts in *ERG*del-positive compared to *ERG*del-negative B-other ALL cases, suggesting that ERG mRNA expression depends on gene-dosage in B-other ALL. However, the *ERG*-related ALL (the “novel”) subgroup still belongs to ALL subtypes with relatively high *ERG* expression, significantly higher than e.g. *MLL*-rearranged, *TCF3-PBX1*-positive or hypodiploid cases as shown by the analysis of publically available data from gene expression profiling. Thus, the *ERG* expression in ALL seems to be regulated also by other mechanisms besides gene-dosage. We have further analyzed *ERG* expression in ALL cases with *ERG*del present at subclonal level and showed that it is comparable to the expression found in *ERG*del-negative B-other ALL and higher than in *ERG*del-positive ALL. In the gene expression profiling and clustering analysis of ALL cases analyzed for *ERG* expression by qRT-PCR the B-other ALL cases with *ERG*del at subclonal level cluster to the same GEP-defined subgroup as cases with *ERG*del present in the entire/major leukemic clone (data not shown). This confirms that the lower *ERG* expression is indeed associated with the reduced *ERG* gene dosage and not with the *ERG*-related ALL subgroup generally and that it does not explain the specific biology of this subgroup.

In their work Tsuzuki et al. showed that particular leukemic cell lines of both myeloid and lymphoid origin including the *ERG*del-positive NALM6 cell line are *ERG*-dependent and stop to proliferate and/or die after its silencing[11]. This suggests that the pro-proliferative/pro-survival function of wild type *ERG* is preserved (at least partially) in cells where one allele is affected by intragenic *ERG* deletion. To the best of our knowledge no cases have been described within *ERG*-related ALL subgroup with biallelic *ERG* deletion. This further supports the hypothesis that wild type *ERG* is important for this leukemia subtype.

In summary, we did not confirm the expression of aberrant proteins from internally deleted *ERG* allele and reduced expression of wild type *ERG* as potential drivers of specific biological and clinical behavior of *ERG*-related ALL subgroup. It was suggested previously that *ERG*del results from illegitimate RAG-mediated recombination potentially facilitated by increased accessibility of *ERG* gene locus in *ERG*-related ALL subgroup [1]. Our findings further favor the hypothesis that *ERG*del indeed represents passenger aberration that is specifically acquired under currently indistinct pre-established biological context and perhaps does not contribute to the final biological character of this subgroup in a substantial manner.

## Supporting Information

S1 File **Supplementary Methods Table A:** PCR primers used for amplification and cloning of *ERG* isoforms **Table B:** PCR primers used to synthesize ERGaberN and ERGaberC templates for T/T assay **Table C:** Primary and secondary antibodies **Table D:** PCR primers and probe used for quantification of physiological *ERG* isoforms **Fig A:** *In silico* analysis of ERGaber reading frame(s) and encoded proteins **Fig B:** Analysis of subcellular localization of *ERG* isoforms by confocal microscopy **Fig C:** ERG protein expression in NALM6 and ALL samples—full scans.
(PDF)

S2 File Raw data of: Fig 2. Translation of ERG isoforms Fig 3. Analysis of subcellular localization of ERG isoforms by western blot Fig 6. Expression of physiological ERG isoforms in ALL subgroups.
(7Z) Click here for additional data file.
