# Supplementary material for: Intragenic ERG Deletions Do Not Explain the Biology of ERG-Related Acute Lymphoblastic Leukemia
Source: PLoS One. 2016 Aug 5;11(8):e0160385. doi: 10.1371/journal.pone.0160385 (PMC4975502; doi:10.1371/journal.pone.0160385)
Supplement: S1 File — Supplementary Methods Table A: PCR primers used for amplification and cloning of ERG isoforms Table B: PCR primers used to synthesize ERGaberN and ERGaberC templates for T/T assay Table C: Primary and secondary antibodies Table D: PCR primers and probe used for quantification of physiological ERG isoforms Fig A: In silico analysis of ERGaber reading frame(s) and encoded proteins Fig B: Analysis of subcellular localization of ERG isoforms by confocal microscopy Fig C: ERG protein expression in NALM6 and ALL samples—full scans. (PDF) [file pone.0160385.s001.pdf]

Intragenic *ERG* deletions do not explain the biology of *ERG*-related acute lymphoblastic leukemia

Eliska Potuckova[1], Jan Zuna[1], Lenka Hovorkova[1], Julia Starkova[1], Jan Stary[2], Jan Trka[1] and Marketa Zaliova[1]

[1]Childhood Leukaemia Investigation Prague (CLIP), Department of Paediatric Haematology and Oncology, 2nd Faculty of Medicine, Charles University and University Hospital Motol, Prague, Czech Republic

[2] Department of Paediatric Haematology and Oncology, 2nd Faculty of Medicine, Charles University and University Hospital Motol, Prague, Czech Republic

Supplementary materials

Supplementary Methods

Cell line cultivation

Cell lines were maintained in Dulbecco´s modified Eagles Medium (HeLa and HEK293T; Thermo Fisher Scientific, Massachusetts, USA) or in Roswell Park Memorial Institute medium (NALM6 and REH; Thermo Fisher Scientific) supplemented with 10% of heat-inactivated Fetal Bovine Serum (FBS; Biosera, France) and Antibiotic-Antimycotic (Thermo Fisher Scientific).

Cloning of *ERG* isoforms

All PCR amplifications were performed using PCR Extender System (5 PRIME GmbH, Germany) according to manufacturer's instructions. PCR products amplified from cDNA were visualized by electrophoresis on 1.5% agarose gel. Selected PCR products were cloned into pCR™4-TOPO® Vector using TOPO® TA Cloning® Kit for Sequencing according to manufacturer's protocol (Thermo Fisher Scientific). Coding sequences of *ERG* isoforms were re-amplified from pCR™4-TOPO® Vector by PCR using primer pairs extended at 5' termini with sequence motifs recognized by XhoI and PstI or EcoRV and XhoI restriction enzymes. PCR products were cut by EcoRV and XhoI restriction enzymes (FastDigest restriction enzymes, Thermo Fisher Scientific) and ligated into pcDNA3.1 vector (kindly provided by Dr. Anthony Ford, Institute of Cancer Research, UK) utilizing T4 DNA Ligase (Thermo Fisher Scientific). Full length coding sequences of successfully cloned inserts were analyzed by Sanger sequencing to exclude clones with PCR-introduced artefacts.

Table A: PCR primers used for amplification and cloning of *ERG* isoforms

Primers used for amplification from cDNA

targeted isoform	primer	primer sequence (5' to 3')
ERG3	forward	GATCGCATTATGGCCAGCAC
ERG2	forward 1	CATGATTCAGACTGTCCCGGAC
ERG2	forward 2	CTGAAGGACATGATTCAGACTGTCC
ERG2	forward 3	CATGATTCAGACTGTCCCGG
ERG2, ERG3	reverse	GCCAGGTCTTTAGTAGTAAGTGCCC

Primers used for cloning from pCR™4-TOPO® into pcDNA3.1

cloned insert	primer	primer sequence (5' to 3')
ERG3, ERG3var, ERGaber	EcoRV-forward	CATGATATCGATCGCATTATGGCCAGCAC
	XhoI-reverse	CATCTCGAGGCCAGGTCTTTAGTAGTAAGTGCCC

Sequences recognized by restriction enzymes are underlined, sequences encoding His and Myc tags are in bold italics

In vitro transcription and translation assay (T/T assay)

Two separate segments of ERGaber coding sequence encoding ERGaberN and ERGaberC were amplified from ERGaber-pcDNA3.1 plasmid by PCR using primers listed in Table B. The synthesized proteins were analyzed by western blot.

Table B: PCR primers used to synthesize ERGaberN and ERGaberC templates for T/T assay

	forward primer 5'to 3'	reverse primer 5'to 3'
ERGaberN	CTCTGTACA***TAATACGACTCACTATAGGG***TTA***CCACC***ATGGCCAGCACTATTAAGGAAGC	GCCTTGAGCCATTCACCTGG
ERGaberC	CTCTGTACA***TAATACGACTCACTATAGGG***TTA***CCACC***ATGGCTCAAGGCAGTGGC	GCCTTGAGCCATTCACCTGG

Forward primers annealing to 5' terminus of particular reading frame (starting with start codon; underlined) were extended at 5'termini to contain T7 promoter sequence (20n motif; bold italics) and Kozak consensus sequence (5n extension of sequence annealing to start codon in 5'direction; bold italics) according to recommendation of kit's producer (Promega).

Transfection of HeLa and HEK293T cells

HeLa and HEK293T cells were seeded on 6 well plate at densities of 480,000 or 600,000 cells per well, respectively, 24 hours before the transfection. Adherent cultures were transfected using Lipofectamine2000 (Thermo Fisher Scientific) reagent according to manufacturer's instructions: 6,4µg plasmid DNA with 8µl of Lipofectamine2000 in 2ml of serum-free medium per well.

Western blot

Protein concentration of nuclear and cytoplasmic protein lysates was determined by Lowry method using DC™ Protein Assay (Bio-Rad, California, USA). Protein lysates and proteins synthesized by T/T assay were diluted in Bolt® LDS Sample Buffer with Bolt® Sample Reducing Agent (Thermo Fisher Scientific) and incubated at 70°C for 10min. Proteins were separated by electrophoresis on Bolt™ 4-12% Bis-Tris Plus Gels (Thermo Fisher Scientific), transferred to nitrocellulose membrane (Bio-Rad) and blocked by Blotting-Grade Blocker (Bio-Rad). Membranes were probed with primary antibodies overnight. Membrane-bound primary antibodies were detected using appropriate secondary antibodies conjugated with horseradish peroxidase (primary and secondary antibodies are listed in Table C). Antibody complexes were visualized using Clarity™ ECL Western Blotting Substrate Kit (Bio-

Rad), SuperSignal™ West Pico Chemiluminescent Substrate Kit and/or SuperSignal™ West Femto Maximum Sensitivity Substrate Kit (Thermo Fisher Scientific) followed by exposition to X-ray films.

Table C: Primary and secondary antibodies

Antibody	*Producer (Cat. No.)*	*Application*	*Dilution*
Anti-ERG antibody (EPR3863)[MC]	Abcam (ab110639)	WB, M	1:1000[WB], 1:250[M]
Erg-1/2/3 Antibody (C-17)[PC]	Santa Cruz (sc-354)	WB	1:1000
Erg-1/2/3 Antibody (C-20)[PC]	Santa Cruz (sc-353)	WB	1:1000
Anti-TATA binding protein TBP antibody[PC]	Abcam (ab63766)	WB	1:1000
Monoclonal Anti-GAPDH antibody produced in mouse[MC]	Sigma-Aldrich (G8795)	WB	1:10000
Goat Anti-Mouse IgG (H + L)-HRP	Bio-Rad (1706516)	WB	1:3000
Goat Anti-Rabbit IgG (H+L)-HRP	Bio-Rad (1706515)	WB	1:4000
Alexa Fluor® 488 Goat Anti-Rabbit IgG(H+L) F(AB)2	Jackson ImmunoResearch (111-546-045)	M	1:500

MC – monoclonal, PC – polyclonal, WB – western blotting, M – confocal fluorescence microscopy

Confocal microscopy

HeLa and HEK293T cells were seeded on sterile cover slips placed inside 6 well plates at densities of 240,000 or 300,000 cells per well, respectively, 24 hours before transfection. The transfection with pcDNA3.1 based *ERG* constructs was performed as described above. Forty-eight hours after transfection cells were fixed by 4% paraformaldehyde (30min), blocked by Normal Goat Serum (Cell Signalling, Massachusetts, USA; 15min), incubated with a primary antibody (60min) and a secondary antibody conjugated with Alexa Fluor® 488 (30min; for antibodies see Table C). Finally, cells were stained by DAPI (Thermo Fisher Scientific) and placed on a microscope slide covered with ProLong® Gold Antifade Reagent (Thermo Fisher Scientific). Microscope slides were inspected using Leica DMi8 inverted microscope equipped with TCS SP8 confocal system and Leica Application Suite X software (Leica Microsystems, Germany). Alexa Fluor® 488 was excited by the 488nm laser and detected in the range of 520-547nm and DAPI was excited by the 405nm laser and detected in the range of 410-452nm.

Quantification of physiological *ERG* isoforms by PCR

All measurements were performed on 2720 Thermal Cycler (Applied Biosystems, USA). Forward primer annealing to exons 9/10 junction and reverse primer annealing to exon 12 were used to quantify expression of *ERG* isoforms containing *ERG* exon 10, while forward primer annealing to exons 9/11 junction and reverse primer annealing to exon 13 were used to quantify expression of *ERG* isoforms lacking *ERG* exon 10. The amplification was carried out in TaqMan® Universal PCR Master Mix (Applied Biosystems, USA) supplemented with primers and probe. Annealing temperature was 63°C for both *ERG* detection systems. Measurements were performed in duplicate. For graphical presentation all expression data were normalized to the lowest expression value within the dataset which was set to 1. Mann-Whitney U test was used to analyze differences in expression between two subgroups.

Table D: PCR primers and probe used for quantification of physiological *ERG* isoforms

primer/probe	5’to 3‘sequence
ERG+10 forward primer	GCATGCTAGAAACACAGGGGGT
ERG+10 reverse primer	GGAAGGAGATGGTTGAGCAGC
ERG-10 forward primer	GGTTAATGCATGCTAGAAACACAGATTTA
ERG-10 reverse primer	CTTGTTGGTCCAAGAATCTGATAAGG
probe*	CGACTGGGGCGTGGGGTGG

ERG+10 = detection system for ERG isoforms containing ERG exon 10; ERG-10 = detection system for ERG isoforms lacking ERG exon 10;
** the identical probe labelled with 6FAM at 5’and TAMRA with 3‘ was used in both ERG detection systems*

Supplementary Figures

A

ATG GCC AGC ACT ATT AAG GAA GCC TTA TCA GTT GTG AGT GAG GAC CAG TCG TTG TTT GAG TGT GCC TAC GGA ACG CCA CAC CTG GCT
AAG ACA GAG ATG ACC GCG TCC TCC TCC AGC GAC TAT GGA CAG ACT TCC AAG ATG AGC CCA CGC GTC CCT CAG CAG GAT TGG CTG TCT
CAA CCC CCA GCC AGG GTC ACC ATC AAA ATG GAA TGT AAC CCT AGC CAG GTG AAT GGC TCA AG GC AGT GGC CAG ATC CAG CTT TGG
CAG TTC CTC CTG GAG CTC CTG TCG GAC AGC TCC AAC TCC AGC TGC ATC ACC TGG GAA GGC ACC AAC GGG GAG TTC AAG ATG ACG GAT
CCC GAC GAG GTG GCC CGG CGC TGG GGA GAG CGG AAG AGC AAA CCC AAC ATG AAC TAC GAT AAG CTC AGC CGC GCC CTC CGT TAC
TAC TAT GAC AAG AAC ATC ATG ACC AAG GTC CAT GGG AAG CGC TAC GCC TAC AAG TTC GAC TTC CAC GGG ATC GCC CAG GCC CTC CAG
CCC CAC CCC CCG GAG TCA TCT CTG TAC AAG TAC CCC TCA GAC CTC CCG TAC ATG GGC TCC TAT CAC GCC CAC CCA CAG AAG ATG AAC
TTT GTG GCG CCC CAC CCT CCA GCC CTC CCC GTG ACA TCT TCC AGT TTT TTT GCT GCC CCA AAC CCA TAC TGG AAT TCA CCA ACT GGG
GGT ATA TAC CCC AAC ACT AGG CTC CCC ACC AGC CAT ATG CCT TCT CAT CTG GGC ACT TAC TAC **TAA**

B

ATG GCC AGC ACT ATT AAG GAA GCC TTA TCA GTT GTG AGT GAG GAC CAG TCG TTG TTT GAG TGT GCC TAC GGA ACG CCA CAC CTG GCT
AAG ACA GAG ATG ACC GCG TCC TCC TCC AGC GAC TAT GGA CAG ACT TCC AAG ATG AGC CCA CGC GTC CCT CAG CAG GAT TGG CTG TCT
CAA CCC CCA GCC AGG GTC ACC ATC AAA ATG GAA TGT AAC CCT AGC CAG GTG AAT GGC TCA *AGG CAG TGG CCA GAT CCA GCT TTG GCA*
GTT CCT CCT GGA GCT CCT GTC GGA CAG CTC CAA CTC CAG CTG CAT CAC CTG GGA AGG CAC CAA CGG GGA GTT CAA GAT GAC GGA TCC
CGA CGA GGT GGC CCG GCG CTG GGG AGA GCG GAA GAG CAA ACC CAA CAT GAA CTA CGA ***TAA*** ...

C

MASTIKEALSVVSEDQSLFECAYGTPHLAKTEMTASSSSDYGQTSKMSPRVPQQDWLSQPPARVTIKMECNPSQVNGSRQWPDPALAVPPGAPVGQLQLQ
LHHLGRHQRGVQDDGSRRGGPALGRAEEQTQHELR-

D

A TGG CCA GCA CTA TTA AGG AAG CCT TAT CAG TTG TGA GTG AGG ACC AGT CGT TGT TTG AGT GTG CCT ACG GAA CGC CAC ACC TGG
CTA AGA CAG AGA TGA CCG CGT CCT CCT CCA GCG ACT ATG GAC AGA CTT CCA AGA TGA GCC CAC GCG TCC CTC AGC AGG ATT GGC TGT
CTC AAC CCC CAG CCA GGG TCA CCA TCA AAA TGG AAT GTA ACC CTA GCC AGG TGA ***ATG*** *GCT CAA G*GC AGT GGC CAG ATC CAG CTT TGG
CAG TTC CTC CTG GAG CTC CTG TCG GAC AGC TCC AAC TCC AGC TGC ATC ACC TGG GAA GGC ACC AAC GGG GAG TTC AAG ATG ACG GAT
CCC GAC GAG GTG GCC CGG CGC TGG GGA GAG CGG AAG AGC AAA CCC AAC ATG AAC TAC GAT AAG CTC AGC CGC GCC CTC CGT TAC
TAC TAT GAC AAG AAC ATC ATG ACC AAG GTC CAT GGG AAG CGC TAC GCC TAC AAG TTC GAC TTC CAC GGG ATC GCC CAG GCC CTC CAG
CCC CAC CCC CCG GAG TCA TCT CTG TAC AAG TAC CCC TCA GAC CTC CCG TAC ATG GGC TCC TAT CAC GCC CAC CCA CAG AAG ATG AAC
TTT GTG GCG CCC CAC CCT CCA GCC CTC CCC GTG ACA TCT TCC AGT TTT TTT GCT GCC CCA AAC CCA TAC TGG AAT TCA CCA ACT GGG
GGT ATA TAC CCC AAC ACT AGG CTC CCC ACC AGC CAT ATG CCT TCT CAT CTG GGC ACT TAC TAC **TAA**

E

MAQGSG**QIQLWQFLLELLSDSSNSSCITWEGTNGEFKMTDPDEVARRWGERKSKPNMNYDKLSRALRYYYDKNIMTKVHGKRYAYKFD**FHGIAQALQP
HPPESSLYKYPSDLPYMGSYHAHPQKMNFVAPHPPALPVTSSSFFAAPNPYWNSPTGGIYPNTRLPTSHMPSHLGTYY-

---

Figure A: In silico analysis of ERGaber reading frame(s) and encoded proteins

(A) Coding sequence of ERGaber (exons 5 and 14 in black, exon 6 in blue) with nucleotides organized into codons corresponding to the cannonical reading frame of physiological transcript variants demonstrating its disruption at exon 6/14 junction (highlighted by green rectangle).
(B) Coding sequence of ERGaber with nucleotides organized into codons of the reading frame following canonical start site. The alternative part of this reading frame is displayed in Italics.
(C) Amino acid sequence of ERGaberN encoded by reading frame following canonical start site. Amino acids encoded by alternative frame are displayed in red.
(D) Coding sequence of ERGaber with nucleotides re-organized into codons preserving the canonical reading frame of exon 14 used in physiological transcript variants. The alternative part of this reading frame (including alternative start codon) is displayed in Italics.
(E) Amino acid sequence of ERGaberC encoded by reading frame following alternative start site. Amino acids encoded by alternative frame are displayed in red. Amino acids constituting ETS domain are in bold.

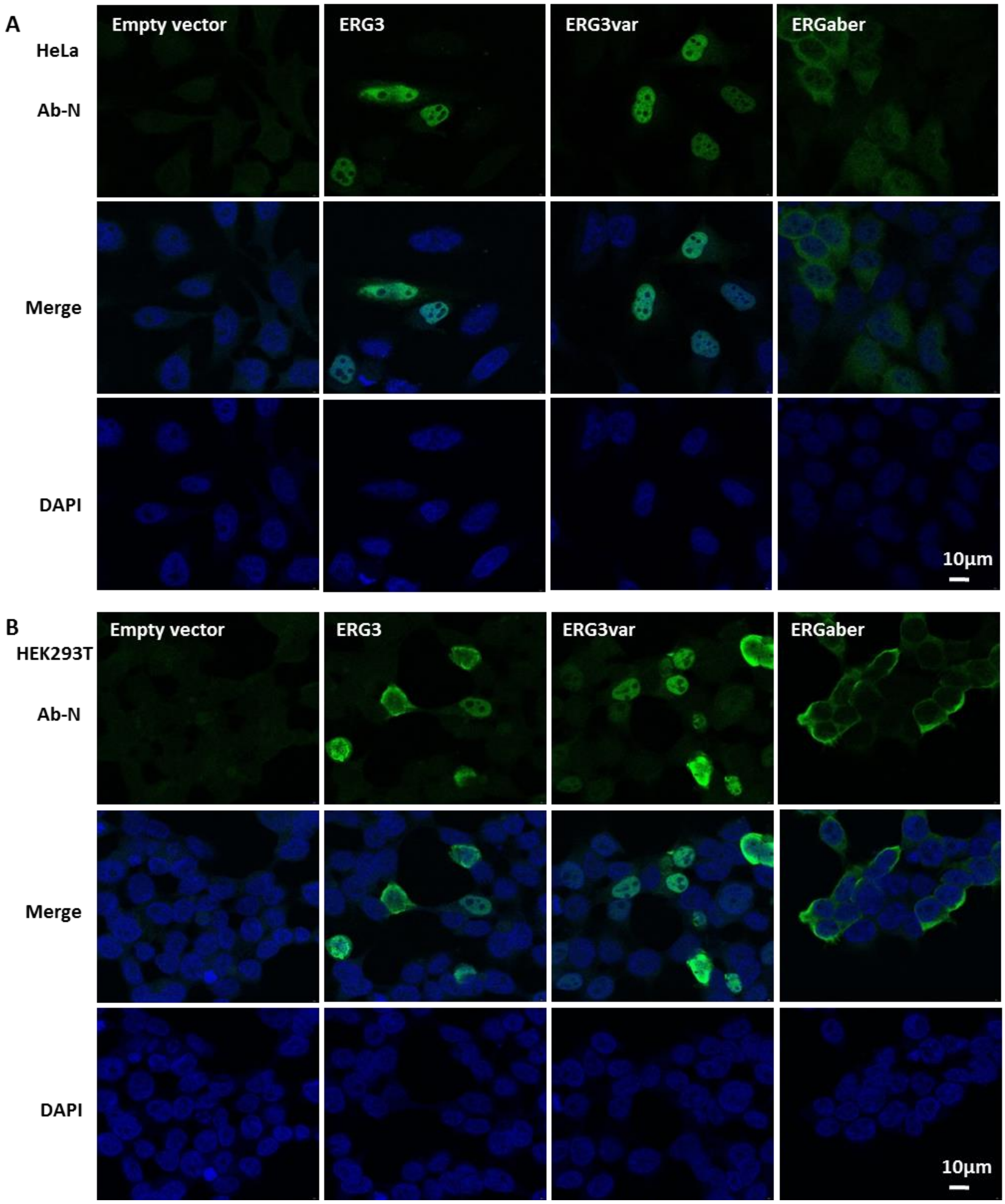

Figure B: Analysis of subcellular localization of *ERG* isoforms by confocal microscopy

HeLa (A) and HEK293T (B) cells were transiently transfected by ERG3, ERG3var and ERGaber isoforms in pcDNA3.1 vector or by empty vector. Forty-eight hours after transfection the presence and subcellular localisation of *ERG* isoforms was analyzed by confocal microscopy using Ab-N antibody. Scale bars represent 10µm.

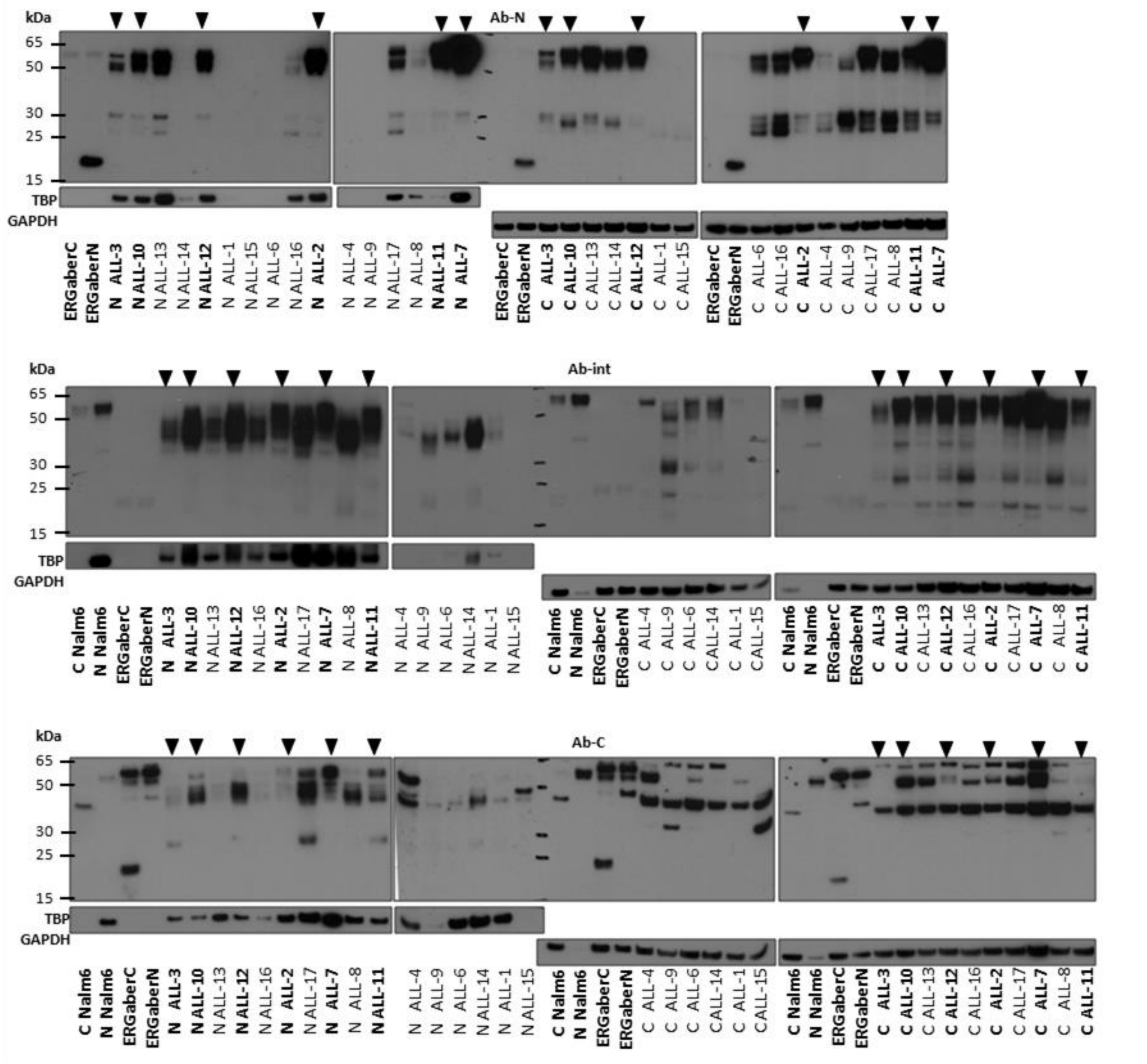

Figure C: ERG protein expression in NALM6 and ALL samples – full scans

Individual lanes (highlighted by black arrows) of these scans (X-ray films from western blot analyses) were cut and grouped and are presented in Figure 5. Remaining lanes were excluded from the analysis because of protein degradation (ALL-14, 1, 15, 6, 16, 4, 9, 8) or subclonality of *ERG*del (*ERG*del positive by PCR but negative by SNParray; ALL-13 and 17).
